# Dengue Virus Envelope Dimer Epitope Monoclonal Antibodies Isolated from Dengue Patients Are Protective against Zika Virus

**DOI:** 10.1128/mBio.01123-16

**Published:** 2016-07-19

**Authors:** J. A. Swanstrom, J. A. Plante, K. S. Plante, E. F. Young, E. McGowan, E. N. Gallichotte, D. G. Widman, M. T. Heise, A. M. de Silva, R. S. Baric

**Affiliations:** a Department of Epidemiology, University of North Carolina at Chapel Hill, Chapel Hill, North Carolina, USA; b Department of Genetics, University of North Carolina at Chapel Hill, Chapel Hill, North Carolina, USA; c Department of Microbiology and Immunology, University of North Carolina at Chapel Hill, Chapel Hill, North Carolina, USA

## Abstract

Zika virus (ZIKV) is a mosquito-borne flavivirus responsible for thousands of cases of severe fetal malformations and neurological disease since its introduction to Brazil in 2013. Antibodies to flaviviruses can be protective, resulting in lifelong immunity to reinfection by homologous virus. However, cross-reactive antibodies can complicate flavivirus diagnostics and promote more severe disease, as noted after serial dengue virus (DENV) infections. The endemic circulation of DENV in South America and elsewhere raises concerns that preexisting flavivirus immunity may modulate ZIKV disease and transmission potential. Here, we report on the ability of human monoclonal antibodies and immune sera derived from dengue patients to neutralize contemporary epidemic ZIKV strains. We demonstrate that a class of human monoclonal antibodies isolated from DENV patients neutralizes ZIKV in cell culture and is protective in a lethal murine model. We also tested a large panel of convalescent-phase immune sera from humans exposed to primary and repeat DENV infection. Although ZIKV is most closely related to DENV compared to other human-pathogenic flaviviruses, most DENV immune sera (73%) failed to neutralize ZIKV, while others had low (50% effective concentration [EC_50_], <1:100 serum dilution; 18%) or moderate to high (EC_50_, >1:100 serum dilution; 9%) levels of cross-neutralizing antibodies. Our results establish that ZIKV and DENV share epitopes that are targeted by neutralizing, protective human antibodies. The availability of potently neutralizing human monoclonal antibodies provides an immunotherapeutic approach to control life-threatening ZIKV infection and also points to the possibility of repurposing DENV vaccines to induce cross-protective immunity to ZIKV.

## IMPORTANCE

ZIKV is an emerging arbovirus that has been associated with severe neurological birth defects and fetal loss in pregnant women and Guillain-Barré syndrome in adults. Currently, there is no vaccine or therapeutic for ZIKV. The identification of a class of antibodies (envelope dimer epitope 1 [EDE1]) that potently neutralizes ZIKV in addition to all four DENV serotypes points to a potential immunotherapeutic to combat ZIKV. This is especially salient given the precedent of antibody therapy to treat pregnant women infected with other viruses associated with microcephaly, such as cytomegalovirus and rubella virus. Furthermore, the identification of a functionally conserved epitope between ZIKV and DENV raises the possibility that a vaccine may be able to elicit neutralizing antibodies against both viruses.

## INTRODUCTION

Zika virus (ZIKV) is an arbovirus in the *Flaviviridae* family, which includes important human pathogens such as Japanese encephalitis virus (JEV), West Nile virus (WNV), yellow fever virus (YFV), and dengue viruses 1 to 4 (DENV-1 to -4) (1). Flaviviruses are traditionally classified as neurovirulent (WNV and JEV) or hemorrhagic (DENV and YFV). ZIKV infection has historically been characterized by self-limiting febrile illness, including mild fever, rash, arthralgia, and conjunctivitis, and was not considered to be a pathogen of major public health concern (2, 3). However, ZIKV caused a large outbreak in Micronesia in 2007 and then throughout Polynesia and the Pacific Islands in 2013 to 2014 (4). In 2015, the first ZIKV outbreak in the Americas was reported in Brazil, where there was no previous evidence of circulation (5). Since then, 46 countries have reported novel outbreaks and ongoing transmission (4). Following the onset of the 2015 outbreak, several groups have identified an association between ZIKV infection and fetal malformations, including spontaneous abortion, intrauterine growth restriction caused by placental insufficiency, and blindness, and a causative link has been associated with microcephaly (2, 4, 6–8). The World Health Organization has also reported an increase in Guillain-Barré syndrome and meningoencephalitis associated with ZIKV (4, 9, 10). The underlying molecular mechanisms driving these severe outcomes remain largely unknown (11).

The emergence of ZIKV overlaps geographically with regions in which DENV is endemic, and ZIKV shares approximately 60% sequence identity with DENV (12). Moreover, multiple dengue vaccine candidates are in phase II and III clinical trials, including a tetravalent vaccine that is currently approved for use in regions where ZIKV is emerging (13–15). Thus, a significant portion of the population in ZIKV outbreak areas has DENV-reactive antibodies, which has complicated ZIKV diagnostics due to cross-reactivity (16). Given the extent to which DENV antibodies are present in the population, is it important to evaluate the possibility of cross-protective neutralizing epitopes that could protect against ZIKV infection. By screening a panel of monoclonal antibodies (MAbs), we found that a class of dengue virus serotype cross-neutralizing MAbs isolated from dengue patients, known as the envelope dimer epitope 1 (EDE1) MAbs, neutralize ZIKV in cell culture and protect from disease in a murine model. A few convalescent-phase immune sera from dengue patients also cross-neutralized ZIKV, further demonstrating the presence of epitopes conserved between ZIKV and DENV that are recognized by human neutralizing antibodies.

## RESULTS

### Neutralization of ZIKV by human, nonhuman primate, and mouse MAbs.

**FIG 1:**
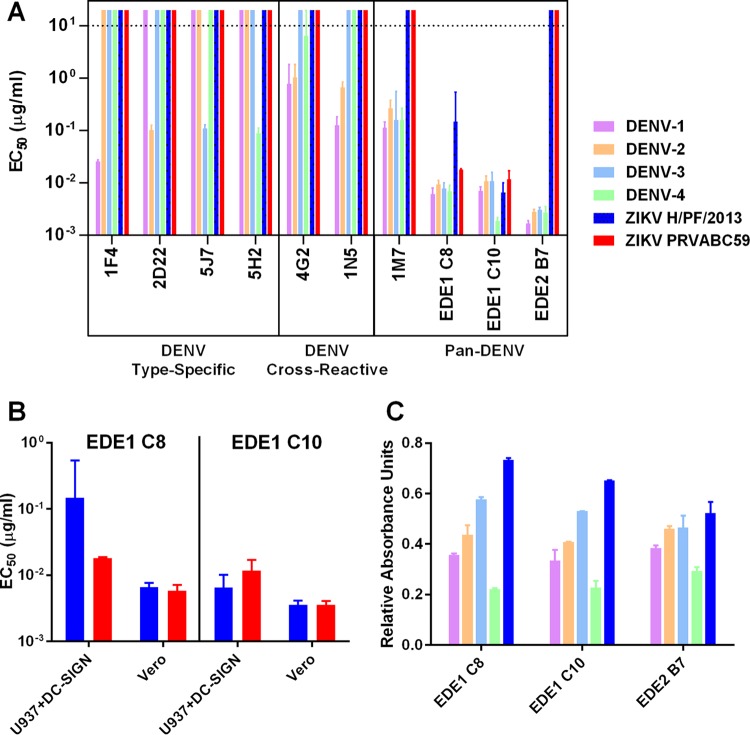
Neutralization and binding of DENV and ZIKV by monoclonal antibodies. (A) MAbs elicited by DENV were evaluated for their ability to neutralize and bind DENV-1, DENV-2, DENV-3, DENV-4, ZIKV H/PF/2013, and ZIKV PRVABC59. (B) The ability of the EDE1 MAbs to neutralize ZIKV H/PF/2013 and ZIKV PRVABD59 was confirmed in both U937+DC-SIGN and Vero cells. (C) Binding of the neutralizing EDE1 MAbs and the nonneutralizing EDE2 B7 MAb to DENV-1, DENV-2, DENV-3, DENV-4, and ZIKV H/PF/2013 was assessed via enzyme-linked immunosorbent assay. Bars for neutralization data in panels A and B represent the means from two replicates with upper and lower 95% confidence intervals. The dotted line indicates the limit of detection for the assay. Nonneutralizing antibodies were assigned a value of twice the limit of detection for visualization. Bars for binding data in panel C represent the mean from two replicates with standard deviations.

To better understand antibody cross-reactivity and functionality between DENV and ZIKV, we tested a large panel of well-characterized human and mouse MAbs for binding and neutralization of two strains of ZIKV: a French Polynesian 2013 strain representing the Asiatic lineage (H/PF/2013) and a strain circulating in the Americas in 2015 (PRVABC59). As expected, human and nonhuman primate type-specific MAbs that strongly neutralize DENV-1 (1F4), DENV-2 (2D22), DENV-3 (5J7), and DENV-4 (5H2) did not neutralize ZIKV (Fig. 1A) (17–20). DENV-cross-reactive MAbs that weakly or moderately neutralized two or more DENV serotypes (4G2, 1N5, and 1M7) also failed to neutralize ZIKV (21, 22). In stark contrast, the potent DENV cross-neutralizing MAbs EDE1 C8 and EDE1 C10 strongly neutralized ZIKV infection of human monocytic cells expressing DC-SIGN (23). ZIKV neutralization by EDE1 C8 and ECE1 C10 was confirmed in Vero cells (Fig. 1B). EDE1 C8 and C10 neutralization of the high-passage-number 1947 Ugandan isolate ZIKV MR766 was also confirmed in Vero cells, with 50% effective concentrations (EC_50_s) of 8.9 × 10^−4^ and 3.4 × 10^−4^ µg/ml, respectively. Interestingly, EDE2 B7 (23), which strongly neutralized all four DENV serotypes, bound but did not neutralize ZIKV (Fig. 1C). Of note, the contact residues of EDE2 B7 are all part of the EDE1 epitope, except for residues 153 to 157, which were too disordered to resolve structurally when bound to DENV-2 (see Table S1 and Fig. S1 in the supplemental material) (24).

**FIG 2:**
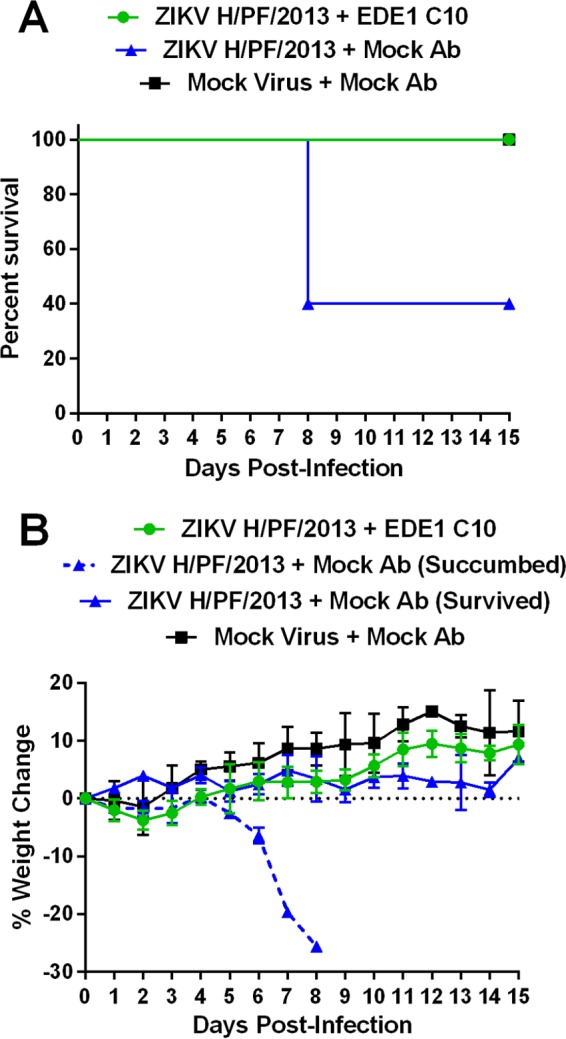
EDE1 C10 protects ZIKV-susceptible mice from infection. Five-week-old type I/II interferon receptor-knockout mice on a C57BL/6 backbone received either EDE1 C10 (*n =* 5) or mock (*n =* 5) treatment and were challenged with 10^2^ FFU of ZIKV H/PF/2013. A mock cohort (*n =* 2) was also included. Survival (A) and weight loss (B) were monitored, and differences between the mock-treated and EDE1 C10-treated cohorts are shown.

Recent studies indicate that flavivirus antibodies that neutralize virus *in vitro* may not necessarily be protective *in vivo* (25). To determine if EDE MAbs protect against ZIKV *in vivo*, a study was performed in type I/II interferon receptor-knockout mice, which develop ZIKV-induced morbidity and mortality (26, 27). The mice were treated with either 10 µg EDE1 C10 or phosphate-buffered saline (PBS) at 1 day preinfection and again at 9 days postinfection and challenged with 10^2^ focus-forming units (FFU) of ZIKV H/PF/2013 or PBS in the footpad. The PBS-treated mice experienced 60% mortality following challenge (Fig. 2A), while the EDE1 C10-protected mice all survived (*P* < 0.05). The EDE1 C10-treated, infected mice largely exhibited no signs of illness, and their weight gain was more than that of mice that were infected with ZIKV and mock antibody but less than that of the mice that were not infected (Fig. 2B).

### Neutralization of ZIKV by convalescent-phase dengue immune sera.

**FIG 3:**
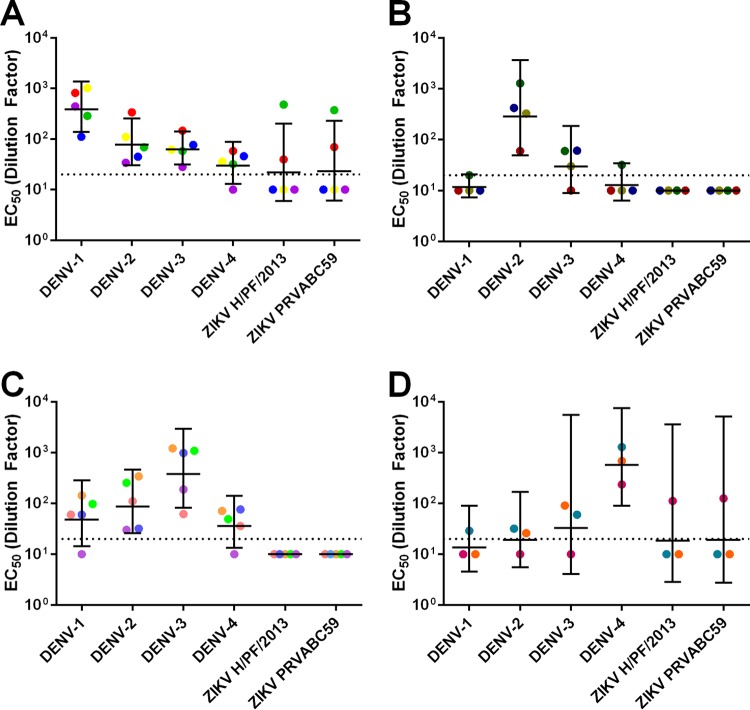
Neutralization of DENV and ZIKV by DENV primary sera. Geometric mean titers of DENV-1 primary sera (A), DENV-2 primary sera (B), DENV-3 primary sera (C), and DENV-4 primary sera (D). Colored points represent individual sera, and horizontal lines represent the geometric mean titers of all sera with upper and lower 95% confidence intervals. The dotted line indicates the limit of detection for the assay. Nonneutralizing sera were assigned a value of one-half of the limit of detection for visualization and calculation of the geometric means and confidence intervals.

People exposed to dengue and other flavivirus infections develop antibodies that change in magnitude and quality over time (28). The ZIKV cross-neutralizing and protective EDE1 C8 and C10 MAbs were derived from plasmablasts collected from individuals a few days after recovery from DENV infections (23). We tested whether convalescent-phase immune sera collected from DENV patients several years after primary or secondary infection contained antibodies that cross-neutralized ZIKV. We tested a panel of 17 serum samples with neutralization profiles consistent with previous exposure to primary DENV-1 (*n* = 5), DENV-2 (*n* = 4), DENV-3 (*n =* 5), and DENV-4 (*n =* 3) infections for cross-neutralization of ZIKV (see Table S2 in the supplemental material). Most of the primary sera failed to cross-neutralize ZIKV. In fact, with primary DENV immune sera, we observed lower levels of ZIKV cross-neutralization than of DENV cross-neutralization (Fig. 3; see also Fig. S2). Notable exceptions to this trend were two primary DENV-1 immune sera and one primary DENV-4 immune serum that contained moderate to high levels of ZIKV-neutralizing antibodies.

**FIG 4:**
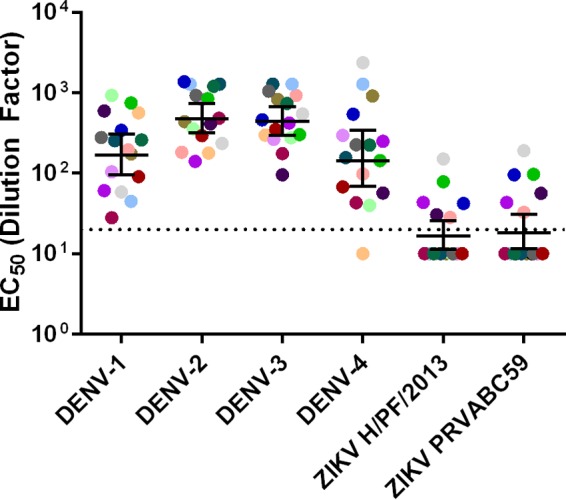
Neutralization of DENV and ZIKV by DENV secondary sera. Geometric mean titers of DENV secondary sera. Colored points represent individual sera, and horizontal lines represent the geometric mean titers of all sera with upper and lower 95% confidence intervals. The dotted line indicates the limit of detection for the assay. Nonneutralizing sera were assigned a value of one-half of the limit of detection for visualization and calculation of the geometric means and confidence intervals.

A hallmark of secondary DENV infections is the induction of dengue virus serotype cross-neutralizing antibodies which reduce the risk of disease from subsequent DENV infections (29). We tested whether convalescent-phase sera from people exposed to secondary DENV infections years previously also cross-neutralized ZIKV. All of the secondary serum samples tested neutralized DENV-1, DENV-2, and DENV-3, and 15 of 16 samples neutralized DENV-4 (Fig. 4; see also Fig. S3 in the supplemental material). There were no statistically significant differences between the DENV serotypes. In contrast, secondary DENV immune sera usually had low or undetectable levels of ZIKV cross-neutralizing antibodies. Six of the 16 sera (38%) had measurable neutralization titers against ZIKV. Five of these six individuals had modest ZIKV neutralization (EC_50_s between 1:20 and 1:100), and only one individual had strong ZIKV neutralization (EC_50_, >1:100). Overall, secondary DENV immune sera poorly neutralized ZIKV compared to cross-neutralization phenotypes noted among other DENV serotypes, and only one individual (6%) strongly cross-neutralized ZIKV.

### Antigenic cartography.

The fact that convalescent-phase DENV immune human sera displayed low cross-neutralization of ZIKV suggests that ZIKV is antigenically distantly related to DENV. To examine the antigenic relationships between ZIKV and DENV, we used antigenic cartography to calculate the Euclidean distances between sera, and metric multidimensional scaling was used to render the data in three dimensions (see Movies S1 and S2 in the supplemental material). Cartography supports the hypothesis that ZIKV is antigenically more distant from DENV-1 to -4 than each DENV serotype is from the others. Moreover, ZIKV-neutralizing sera did not have universally higher DENV-neutralizing titers than ZIKV-nonneutralizing sera. Indeed, cartography suggests that neutralization titers of primary and secondary sera across all four DENV strains do not predict cross-neutralization outcomes with ZIKV, suggesting that these cross-neutralizing antibodies represent a rare subset of anti-DENV antibodies that develop in a subset of individuals within a population.

## DISCUSSION

Because ZIKV cocirculates with other flaviviruses, especially the four DENV serotypes, an understanding of the antigenic relationships between ZIKV and other flaviviruses and how these interactions modulate ZIKV replication, disease, and transmission is imperative. Among the pathogenic human flaviviruses, ZIKV is most closely related to DENV, and the goal of this study was to identify any shared epitopes between DENV and ZIKV targeted by cross-protective human antibodies (12). Primary DENV infections induce serotype-specific neutralizing and protective antibody responses, whereas repeat DENV infections lead to the induction of serotype cross-neutralizing and cross-protective responses (28, 30). We assessed the long-term immunological cross-reactivity of DENV sera with ZIKV using panels of MAbs and immune sera from people exposed to DENV. Neutralization assays with multiple type-specific and cross-reactive MAbs identified a single set of MAbs in our panel that could neutralize ZIKV and protect against lethal infection *in vivo*: EDE1 C8 and EDE1 C10. While we recognize that a broader set of human MAbs might identify novel cross-neutralizing epitopes conserved in ZIKV and DENV, the EDE1 antibodies potently neutralized a French Polynesian 2013 strain representing the Asiatic lineage (H/PF/2013) and, importantly, a strain circulating in the Americas in 2015 (PRVABC59). Indeed, the dose of EDE1 C10 administered to protect *in vivo* (two doses of 10 µg) is far less than the 500 µg required for the fusion loop-targeting mouse MAb 2A10G6 (31). Thus, it seems likely that these EDE1 MAbs will prove efficacious against multiple ZIKV strains *in vivo*. Consonant with this hypothesis, an alignment of the EDE1 contact residues on DENV as previously identified by X-ray crystallography and ZIKV reveals considerable conservation among contact residues between all four DENV serotypes and ZIKV, readily explaining the cross-neutralization phenotypes noted in our studies (24).

The EDE2 B7 MAb did not neutralize ZIKV despite significant epitope overlap with the EDE1 antibodies. EDE2 B7 is reported to be sensitive to the glycan at position 153 of the DENV envelope protein; ZIKV has a glycan at position 154, but the amino acid insertion in the glycan loop may alter the presentation of the glycan (23, 32). Moreover, EDE1 antibodies reach further into domains I and III, providing an additional structural framework for robust binding that may not be as strongly impacted by the insertion in the glycan loop. These data suggest that the EDE1 epitope may be critical to eliciting antibodies that protect against both DENV and ZIKV and that efforts to develop vaccines and therapeutics should emphasize this population of antibodies.

The initial description of EDE antibodies reported that they were immunodominant in nearly half of the study’s subjects (including one individual with a primary DENV infection), but there are several important caveats: the study had a small cohort (*n =* 7) and the frequency of EDE antibodies was not broken down into EDE1 versus EDE2 (23). Additionally, the EDE antibodies were isolated from circulating plasmablasts elicited early during the first couple of weeks following a confirmed DENV infection in Southeast Asia. It is unclear whether these EDE-expressing plasmablasts are frequently elicited across the global population, whether they are dependent on select sequential strain infection serotype patterns, and whether these plasmablasts mature into long-lived memory B cells or are lost. Nor is it known whether the level of EDE antibody expression in the circulating short- and long-term serological repertoire is sufficiently robust to protect from repeat infections. For example, while highly cross-neutralizing monoclonal antibodies can be elicited against the GII.4 human epidemic noroviruses, only a few percent of the human population actually produces these potent antibodies (33). Clearly, new diagnostic metrics such as epitope swap viruses, antibody depletion assays, and blockade of binding assays are needed to evaluate the levels of EDE antibodies in polyclonal sera after primary and secondary infection (34–37).

Some, but not all, DENV primary and secondary immune sera are capable of cross-neutralizing ZIKV. The limited cross-neutralization of ZIKV by DENV primary sera is likely attributed to the mostly type-specific long-term response that follows a single DENV infection (28). After secondary infection, DENV-elicited antibody responses are thought to maintain the type-specific response while simultaneously generating more broadly neutralizing antibodies that typically protect from further DENV infection with any serotype (30). Surprisingly, we observed no cross-neutralization of ZIKV in many individuals who had broadly cross-neutralizing antibodies to three or more DENV serotypes. We conclude that, despite the close phylogenetic relationship of DENV and ZIKV, durable long-lived antibody immune responses that broadly cross-neutralize DENV serotypes are usually not effective against ZIKV. What we did observe were clear cases of ZIKV cross-neutralization in a minority of subjects with DENV type-specific or cross-neutralizing antibody responses. The molecular basis of why some dengue-immune individuals cross-neutralize ZIKV is currently not known. Possible explanations for cross-neutralization include previous exposure to both DENV and ZIKV or the presence of EDE1 or related antibody classes in a subset of individuals. We propose that EDE1-like antibodies are, at least in part, responsible for cross-neutralizing activity in immune sera.

Gamma globulin treatment of pregnant women infected with rubella virus is associated with a reduction in harmful outcomes in the fetus (38). Similar therapies have had mixed success in preventing cytomegalovirus-driven birth defects, and immunotherapeutic human monoclonal antibody clinical trials are still ongoing (39–41). Thus, it is reasonable to assume that human monoclonal antibody therapy may be a viable treatment option to protect the developing fetus in pregnant women infected with ZIKV. Although additional therapeutic studies during infection and pregnancy will be required, the identification of an epitope that neutralizes ZIKV *in vitro* and *in vivo* represents a significant first step toward preventing ZIKV-driven fetal malformation and loss. Furthermore, the fact that the same antibodies targeting EDE1 are able to strongly neutralize both DENV and ZIKV is highly desirable, as diagnostic tests cannot always rapidly and reliably differentiate between the two infections. The strongly cross-neutralizing phenotypes of EDE1 C8 and EDE1 C10 should reduce the likelihood that a DENV patient who has been misdiagnosed with a ZIKV infection would experience disease enhancement after treatment with an EDE1 therapeutic antibody. In some individuals, the EDE1-like antibody may also be elicited by the existing tetravalent dengue vaccines already in late-stage clinical trials or available on the market. Further testing is required to determine the frequency of this antibody in the population following both natural infection and vaccination.

## MATERIALS AND METHODS

### Cells and viruses.

All viruses were propagated in C6/36 *Aedes albopictus* cells as previously described. C6/36 cells were grown in minimal essential medium (Gibco, Grand Island, NY) at 32°C. Vero-81 cells were grown in Dulbecco’s modified Eagle’s medium (Gibco, Grand Island, NY), and U937+DC-SIGN cells were maintained in RPMI 1640 (Gibco, Grand Island, NY) at 37°C. All media were supplemented with 10% (Vero-81) or 5% (C6/36 and U937+DC-SIGN) fetal bovine serum (HyClone, Logan, UT), 0.1 mM nonessential amino acids (Gibco, Grand Island, NY), and 100 U/ml penicillin and 100 mg/ml streptomycin (Gibco, Grand Island, NY). U937+DC-SIGN medium was additionally supplemented with 2 mM GlutaMAX (Gibco, Grand Island, NY), 10 mM HEPES (Cellgro, Manassas, VA), and 2-mercaptoethanol (Sigma, St. Louis, MO). All cells were incubated in the presence of 5% CO_2_.

ZIKV H/PF/2013 viral stocks were obtained from Michael S. Diamond (Washington University, St. Louis, MO). ZIKV PRVABC59 viral stocks were obtained from the Centers for Disease Control and Prevention (Atlanta, GA). DENV strains used in the polyclonal neutralization panel (DENV-1 WestPac74, DENV-2 S-16803, DENV-3 CH-53489, and DENV-4 TVP-376) were obtained from natural isolates maintained in the laboratory of Aravinda M. de Silva. DENV strains used in the monoclonal antibody panel (DENV-1 WestPac74, DENV-2 S-16803, DENV-3 UNC3001, and DENV-4 SriLanka 92A) were obtained from infectious clones in the laboratory of Ralph S. Baric (34, 35).

### Serum and antibodies.

Deidentified human DENV immune sera and plasma were collected from individuals with naturally acquired DENV infections confirmed via serology. All donations were collected in compliance with the Institutional Review Board of the University of North Carolina at Chapel Hill (protocol 08-0895). Deidentified human immune sera previously collected from the Pediatric Dengue Vaccine Initiative were also used.

Monoclonal antibodies were purified from hybridomas (1M7 and 1N5) or synthetically generated by Lake Pharma (Belmont, CA) from published sequences (1F4, 2D22, 5J7, 5H2, 4G2, EDE1 C8, EDE1 C10, and EDE2 B7); the latter are available upon request.

### *In vitro* neutralization.

Human sera or monoclonal antibodies were serially diluted 3-fold and mixed with sufficient virus to cause 15% infection in U937+DC-SIGN cells. Dilution medium contained reduced (2%) fetal bovine serum and was supplemented with 2 mM CaCl_2_ and MgCl_2_. The virus-antibody mixtures were incubated for 45 min in a 96-well plate at 37°C. Following this incubation, 5 × 10^4^ cells were added and the infection was allowed to proceed for 2 h at 37°C. The volume of medium in each well was increased to 200 µl, and the cells were returned to 37°C for a total of 24 h. After 24 h, the cells were fixed in paraformaldehyde, permeabilized, blocked with normal mouse serum in permeabilization buffer, and stained with Alexa Fluor 488-conjugated (Molecular Probes, Eugene, OR) 4G2 antibody. Unbound antibody was washed off, and cells were resuspended in Hanks’ buffered salt solution (Gibco, Grand Island, NY) supplemented with 2% fetal bovine serum. Assays were performed twice and in duplicate. Samples were read on a Guava easyCyte 5HT flow cytometer (Millipore) as previously described by our group (42).

Neutralization in Vero-81 cells was assessed by serially diluting the monoclonal antibodies 10-fold and mixing with approximately 150 focus-forming units of virus. Dilution medium contained reduced (2%) fetal bovine serum. The virus-antibody mixtures were incubated for 1 h in a 96-well plate at 37°C and then transferred to a monolayer of Vero-81 cells in a 96-well plate. Following a further 1-h incubation at 37°C, the monolayers were overlaid with Opti-MEM (Gibco, Grand Island, NY) containing 2% fetal bovine serum and 1% (wt/vol) carboxymethyl cellulose (Sigma, St. Louis, MO). Infected plates were incubated for 2 days at 37°C with 5% CO_2_, at which time they were fixed with paraformaldehyde, permeabilized, blocked with normal goat serum (Sigma, St. Louis, MO) in permeabilization buffer, stained with 4G2 primary antibody followed by secondary horseradish peroxidase (HRP)-conjugated anti-mouse IgG (KPL, Gaithersburg, MD), washed again, and developed with TrueBlue peroxidase substrate (KPL, Gaithersburg, MD).

### Binding assays.

High-binding Microlon 600 96-well plates (VWR, Radnor, PA) were coated with 100 ng of 4G2 and 2H2 antibody in 0.1 M carbonate buffer, pH 9.6, overnight at 4°C. Unbound antibody was rinsed with wash buffer (Tris-buffered saline [TBS] plus 0.2% Tween), and wells were coated with blocking buffer (TBS plus 0.05% Tween) for 1 h at 37°C. Virus was diluted in blocking buffer at a concentration sufficient to result in approximately equal reactivity with a human cross-reactive control serum and was added to the plate for 1 h at 37°C. Unbound virus was rinsed in wash buffer, and 1 µg of each primary antibody (or the control serum) diluted in blocking buffer was added to the plate for 1 h at 37°C. Unbound primary antibody was rinsed in wash buffer, and alkaline phosphatase-conjugated goat anti-human IgG antibody (Sigma, St. Louis, MO) at 1:2,500 in blocking buffer was added to the plate for 1 h at 37°C. Unbound secondary antibody was rinsed in wash buffer, the plate was developed with SigmaFast *p*-nitrophenyl phosphate tablets (Sigma, St. Louis, MO), and signal was read at 405 nm.

### Animal studies.

Cohorts of five (virus only and virus with antibody) or two (mock) 5-week-old type I/II interferon receptor-knockout mice (Ifnar^−/−^ and Ifngr^−/−^) on a C57BL/6 backbone were used in a single experiment. On days −1 and 9 postinfection, mice received either PBS (mock and virus only) or 10 µg EDE1 C10 antibody (virus with antibody) in a 100-µl dose delivered intraperitoneally. On day 0, mice received either PBS (mock) or 100 FFU of ZIKV H/PF/2013 (virus only and virus with antibody) in a 10-µl dose delivered subcutaneously in the hind left footpad (27). Mice were monitored daily for 14 days postinfection for weight loss and signs of illness. Mice were humanely euthanized if they became moribund and counted as deceased for that day. All work was performed in adherence to the *Guide for the Care and Use of Laboratory Animals* (43).

### Antigenic cartography.

Antigenic cartography was performed using the EC_50_s generated from the neutralization assays with DENV-1, -2, -3, and -4 in U937+DC-SIGN cells. The data were normalized as described in the work of Cai et al. (44). Euclidean distances between sera were calculated, and metric multidimensional scaling was used to render the data in three dimensions (45). All calculations and images were generated in R Studio, version 0.99.467 (RStudio Inc., Boston, MA). Movie files were rendered using Adobe Photoshop software (Adobe, San Jose, CA).

### Statistical analysis.

When analyzing neutralization assays, antibody and serum concentrations were log_10_ transformed. Next, the EC_50_s were calculated using the sigmoidal dose response (variable slope) equation of Prism 6 (GraphPad Software, La Jolla, CA). Reported values were required to have: at least 5,000 recorded events per data point (in the case of the U937+DC-SIGN assay), an *R*-squared value of greater than 0.75, a Hill slope value of at least 0.7 for monoclonal antibodies and 0.5 for sera, and an EC_50_ within the range of the assay. Variation between groups was measured by one-way analysis of variance (ANOVA) with a Bonferroni *post hoc* test. *P* values of less than 0.05 were considered statistically significant.

Absorbance signals for each virus group in the binding assay were multiplied or divided such that the signal for that virus against the common control serum was set to 1. Each assay was run singly with technical duplicates. Means and standard deviations were calculated in Prism 6 (GraphPad Software, La Jolla, CA).

Survival rates in the animal experiment were analyzed using the log rank (Mantel-Cox) test in Prism 6 for Windows (GraphPad Software, La Jolla, CA). The virus-with-antibody cohort was compared to the virus-only cohort.

## SUPPLEMENTAL MATERIAL

Figure S1  EDE1 and EDE2 epitopes on ZIKV envelope. Location of the EDE1 (C8) (A), EDE1 C10 (B), and EDE2 B7 (C) epitopes on the ZIKV virion. Color coding is based on the infectious clones used in the text with ZIKV H/PF/2013 and ZIKV PRVABC59 (accession numbers KJ776791.1 and KU501215.1, respectively). Contact residues are visualized as spheres, and disordered residues are visualized as sticks (24). Images were generated in PyMOL using the structure 5IZ7 (32). Download Figure S1, TIF file, 2.8 MB

Figure S2  Neutralization curves of DENV primary sera with ZIKV H/PF/2013. Neutralization curves for primary DENV-1 (A), DENV-2 (B), DENV-3 (C), and DENV-4 (D) sera against ZIKV H/PF/2013 in a U937+DC-SIGN assay. Download Figure S2, TIF file, 0.1 MB

Figure S3  Neutralization curves of DENV secondary sera with ZIKV H/PF/2013. Neutralization curves for all DENV secondary sera against ZIKV H/PF/2013 in a U937+DC-SIGN assay. Download Figure S3, TIF file, 0.1 MB

Movie S1  Three-dimensional antigenic cartography of DENV primary and secondary immune sera. Antigenic cartography of all sera based upon the neutralization of DENV-1, -2, -3, and -4. Sera are colored according to either their DENV reactivity classification (primary DENV-1, primary DENV-2, etc.) (left) or their ability to neutralize ZIKV (right). Download Movie S1, MP4 file, 0.1 MB

Movie S2  Three-dimensional antigenic cartography of DENV secondary immune sera. Antigenic cartography of all DENV secondary sera based upon the neutralization of DENV-1, -2, -3, and -4. Sera are colored according to their ability to neutralize ZIKV. Note that among the secondary sera, there is no distinct clustering of ZIKV-neutralizing versus ZIKV-nonneutralizing sera. Download Movie S2, MP4 file, 0.1 MB

Table S1  Amino acid diversity among DENV and ZIKV strains in EDE1 and EDE2 contact residues. Sequence alignment of EDE MAb contact residues. DENV envelope sequences are from the infectious clones used in the text with ZIKV H/PF/2013 and ZIKV PRVABC59 (accession numbersKJ776791.1 and KU501215.1, respectively). Numbering is based on sequence of ZIKV envelope. Yes, identified contact residue; ?, contact status unknown (structure was too disordered to make a determination) (24). Table S1, DOCX file, 0.1 MB

Table S2  Characteristics of human plasma and sera used in neutralization assays. List of the human serum samples with the location of infection and, if known, the approximate date of the infection and sample collection. Table S2, DOCX file, 0.1 MB
