# Supplementary figures and images for: Dengue Virus Envelope Dimer Epitope Monoclonal Antibodies Isolated from Dengue Patients Are Protective against Zika Virus
Source: mBio. 2016 Jul 19;7(4):e01123-16. doi: 10.1128/mBio.01123-16 (PMC4958264; doi:10.1128/mBio.01123-16)

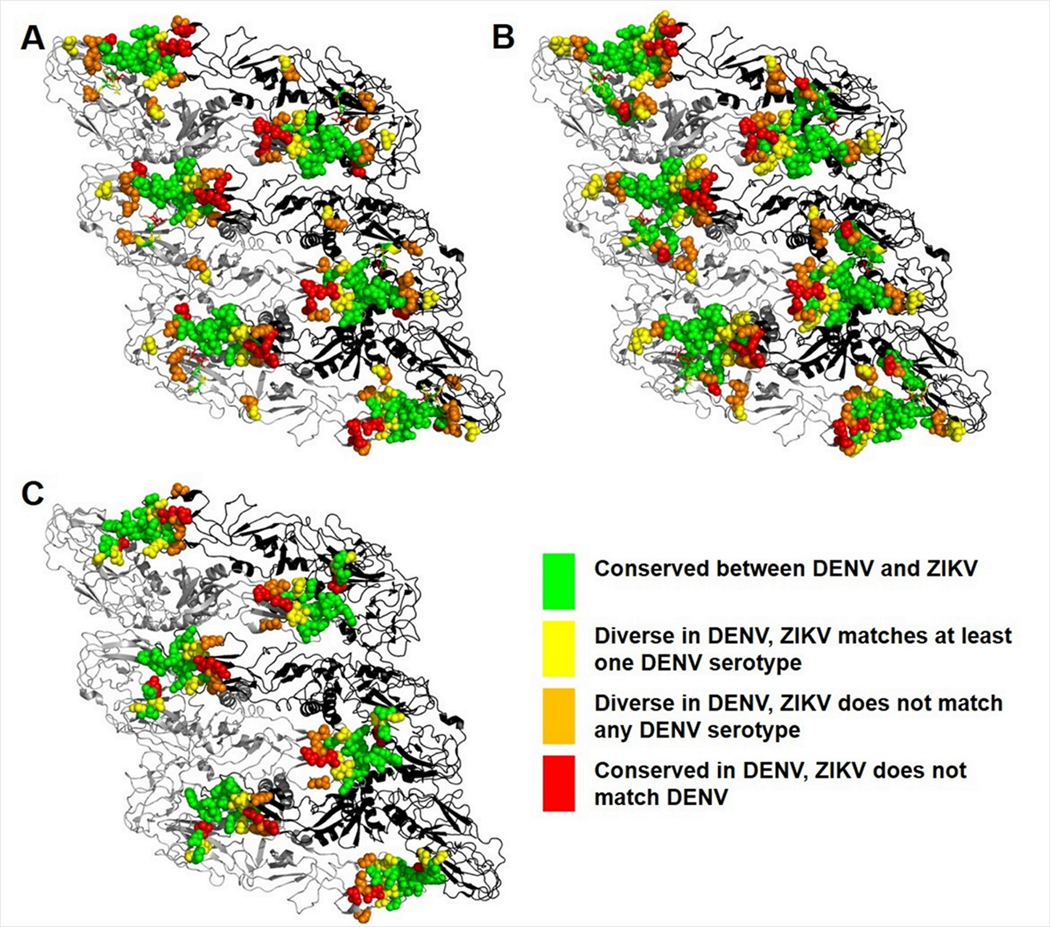

Supplement: Figure S1 — EDE1 and EDE2 epitopes on ZIKV envelope. Location of the EDE1 (C8) (A), EDE1 C10 (B), and EDE2 B7 (C) epitopes on the ZIKV virion. Color coding is based on the infectious clones used in the text with ZIKV H/PF/2013 and ZIKV PRVABC59 (accession numbers KJ776791.1 and KU501215.1, respectively). Contact residues are visualized as spheres, and disordered residues are visualized as sticks (24). Images were generated in PyMOL using the structure 5IZ7 (32). Download [file mbo004162914sf1.tif]
