# Supplementary material for: Dengue Virus Envelope Dimer Epitope Monoclonal Antibodies Isolated from Dengue Patients Are Protective against Zika Virus
Source: mBio. 2016 Jul 19;7(4):e01123-16. doi: 10.1128/mBio.01123-16 (PMC4958264; doi:10.1128/mBio.01123-16)
Supplement: Table S1 — Amino acid diversity among DENV and ZIKV strains in EDE1 and EDE2 contact residues. Sequence alignment of EDE MAb contact residues. DENV envelope sequences are from the infectious clones used in the text with ZIKV H/PF/2013 and ZIKV PRVABC59 (accession numbersKJ776791.1 and KU501215.1, respectively). Numbering is based on sequence of ZIKV envelope. Yes, identified contact residue; ?, contact status unknown (structure was too disordered to make a determination) (24). [file mbo004162914st1.docx]

| Position | N-Linked  Glycosylation | DENV-1 | DENV-2 | DENV-3 | DENV-4 | ZIKV  H/PF/2013 | ZIKV  PRVABC59 | EDE1  C8 | EDE1  C10 | EDE2  B7 |
| --- | --- | --- | --- | --- | --- | --- | --- | --- | --- | --- |
| 2 |  | R | R | R | R | R | R |  | Yes |  |
| 27 |  | H | H | H | H | H | H |  | Yes |  |
| 28 |  | G | G | G | G | G | G |  | Yes |  |
| 44 |  | E | E | E | E | E | E |  | Yes |  |
| 45 |  | L | L | L | L | L | L |  | Yes |  |
| 46 |  | L | I | Q | T | V | V |  | Yes |  |
| 47 |  | K | K | K | K | T | T |  | Yes |  |
| 67 | Yes (DENV Only) | N | N | N | N | D | D | Yes | Yes | Yes |
| 68 |  | T | T | I | I | M | M | Yes | Yes | Yes |
| 69 |  | T | T | T | T | A | A | Yes | Yes | Yes |
| 70 |  | T | T | T | T | S | S | Yes | Yes | Yes |
| 71 |  | D | E | D | A | D | D | Yes | Yes | Yes |
| 72 |  | S | S | S | T | S | S | Yes | Yes | Yes |
| 73 |  | R | R | R | R | R | R | Yes | Yes | Yes |
| 74 |  | C | C | C | C | C | C | Yes | Yes | Yes |
| 77 |  | Q | Q | Q | Q | Q | Q | Yes | Yes |  |
| 81 |  | T | S | V | Y | Y | Y |  | Yes |  |
| 82 |  | L | L | L | L | L | L |  | Yes |  |
| 83 |  | V | N | P | K | D | D | Yes | Yes | Yes |
| 84 |  | E | E | E | E | K | K | Yes | Yes |  |
| 97 |  | V | V | V | V | V | V | Yes | Yes | Yes |
| 98 |  | D | D | D | D | D | D | Yes | Yes | Yes |
| 99 |  | R | R | R | R | R | R | Yes | Yes | Yes |
| 101 |  | W | W | W | W | W | W | Yes | Yes | Yes |
| 102 |  | G | G | G | G | G | G | Yes | Yes | Yes |
| 103 |  | N | N | N | N | N | N | Yes | Yes | Yes |
| 104 |  | G | G | G | G | G | G | Yes | Yes | Yes |
| 105 |  | C | C | C | C | C | C | Yes | Yes |  |
| 106 |  | G | G | G | G | G | G | Yes | Yes |  |
| 113 |  | L | I | L | V | L | L | Yes | Yes | Yes |
| 115 |  | T | T | T | T | T | T |  | Yes |  |
| 148 |  | Q | E | Q | T | H | H | Yes |  |  |
| 151 |  | Q | A | Q | A | M | M | ? | ? |  |
| 152 |  | V | V | V | V | I | I | ? | ? |  |
| 153 |  | G | G | G | G | V | V | ? | ? | Yes |
| 154 | Yes (DENV & ZIKV) | N | N | N | N | N | N | ? | ? | Yes |
| 155 |  | E | D | E | D | D | D | ? | ? | Yes |
| 156 |  | T | T | T | T | T | T | ? | ? | Yes |
| 157 |  | T | G | - | S | G | G | ? | ? | Yes |
| 163 |  | H | H | Q | H | N | N | Yes |  |  |
| 251 |  | K | K | K | K | K | K | Yes | Yes | Yes |
| 252 |  | K | K | K | R | R | R | Yes | Yes | Yes |
| 253 |  | Q | Q | Q | Q | Q | Q | Yes | Yes | Yes |
| 254 |  | E | D | E | D | T | T | Yes | Yes | Yes |
| 276 |  | Q | Q | Q | D | E | E |  | Yes |  |
| 278 |  | S | S | S | G | D | D | Yes | Yes |  |
| 279 |  | G | S | G | D | G | G | Yes | Yes |  |
| 315 |  | E | V | K | D | T | T | Yes | Yes |  |
| 316 |  | K | K | K | K | K | K | Yes | Yes |  |
| 317 |  | E | E | E | E | I | I | Yes |  |  |
| 329 |  | Q | R | K | K | E | E | Yes | Yes |  |
| 331 |  | K | Q | E | K | Q | Q |  | Yes |  |
| 371 |  | E | D | E | N | N | N | Yes | Yes |  |
