# Supplementary material for: Dengue Virus Envelope Dimer Epitope Monoclonal Antibodies Isolated from Dengue Patients Are Protective against Zika Virus
Source: mBio. 2016 Jul 19;7(4):e01123-16. doi: 10.1128/mBio.01123-16 (PMC4958264; doi:10.1128/mBio.01123-16)
Supplement: Table S2 — Characteristics of human plasma and sera used in neutralization assays. List of the human serum samples with the location of infection and, if known, the approximate date of the infection and sample collection. [file mbo004162914st2.docx]

| Serum Classification | Serum ID | Location | Year of Infection | Year of Collection |
| --- | --- | --- | --- | --- |
| Primary DENV-1 | DT145 | South America | 2005 | Spring 2016 |
| Primary DENV-1 | DT147 | South America | Fall 2015 | Spring 2016 |
| Primary DENV-1 | DT153 | South America | 2010 | End 2014 |
| Primary DENV-1 | SS05/262 | Asia | Unknown | Unknown |
| Primary DENV-1 | SS99/1230 | Asia | Unknown | Unknown |
| Primary DENV-2 | DT001 | Asia | 1996 | Spring 2016 |
| Primary DENV-2 | DT155 | South America | 2000 | Spring 2015 |
| Primary DENV-2 | SS08/90 | Asia | Unknown | Unknown |
| Primary DENV-2 | SS08/91 | Asia | Unknown | Unknown |
| Primary DENV-3 | DT115 | Asia | Unknown | Winter 2009 |
| Primary DENV-3 | DT118 | South America | 2009 | Fall 2010 |
| Primary DENV-3 | DT125 | South America | 2007 | Spring 2012 |
| Primary DENV-3 | DT133 | South America | 2002 | Summer 2012 |
| Primary DENV-3 | SS06/297 | Asia | Unknown | Unknown |
| Primary DENV-4 | GS0239 | Unknown | Unknown | Unknown |
| Primary DENV-4 | SS06/105 | Asia | Unknown | Unknown |
| Primary DENV-4 | SS06/302 | Asia | Unknown | Unknown |
| Secondary DENV | DT000 | Asia | <1982 | Spring 2016 |
| Secondary DENV | DT112 | South America | 2001 | Spring 2009 |
| Secondary DENV | DT116 | Asia | Unknown | Fall 2009 |
| Secondary DENV | DT141 | South America | 2006 | Fall 2014 |
| Secondary DENV | DT146 | Asia | 2013 | Fall 2014 |
| Secondary DENV | DT160 | South America | 2015 | Summer 2015 |
| Secondary DENV | DV003 | Asia | Unknown | Unknown |
| Secondary DENV | DT144 | Asia | Unknown | Unknown |
| Secondary DENV | SS06/123 | Asia | Unknown | Unknown |
| Secondary DENV | SS06/124 | Asia | Unknown | Unknown |
| Secondary DENV | SS06/125 | Asia | Unknown | Unknown |
| Secondary DENV | SS09/157 | Asia | Unknown | Unknown |
| Secondary DENV | SS09/159 | Asia | Unknown | Unknown |
| Secondary DENV | SS09/165 | Asia | Unknown | Unknown |
| Secondary DENV | SS09/250 | Asia | Unknown | Unknown |
| Secondary DENV | SS09/251 | Asia | Unknown | Unknown |
